# Comparison of anti-peritoneal fibrotic effects between an mTORC1-specific blocker and a PI3K/mTOR dual-blocker

**DOI:** 10.1080/0886022X.2019.1596818

**Published:** 2019-04-15

**Authors:** Tian Xu, Tao Lin, Jingyuan Xie, Hong Ren, Nan Chen, Weiming Wang

**Affiliations:** Department of Nephrology, Institute of Nephrology, Ruijin Hospital, Shanghai Jiaotong University School of Medicine, Shanghai, China

**Keywords:** Peritoneal fibrosis, mTOR, PI3K, Rapamycin, BEZ235

## Abstract

**Objective:** To compare the anti-peritoneal fibrotic effects between a mammalian target of rapamycin complex 1-specific blocker and a phosphatidyl-inositol 3-kinase/mammalian target of rapamycin dual-blocker.

**Methods:** A total of 40 male Sprague-Dawley rats were randomly divided into five groups with eight animals per group. The normal group (N group) did not receive any intervention. The normal saline group (NS group) received an intraperitoneal injection of normal saline at 1 ml/100 g daily. The model group (3 W group), rapamycin (RAPA) group and BEZ235 (PI3K/mTOR dual-blocker) group all received an intraperitoneal injection of 0.1% chlorhexidine gluconate at 1 ml/100g daily. And the RAPA and BEZ235 groups also received a 0.5 mg/d RAPA or 2.5 mg/d BEZ235 gavage every day, respectively. Rats in each group were sacrificed after 3 weeks.

**Results:** Immunohistochemistry, real-time PCR and western blotting analysis of fibrosis-related indicators (FN, Col 1, and α-SMA) confirmed that RAPA and BEZ235 significantly inhibited peritoneal fibrosis and that these two drugs had similar effects. The p-Akt, p-mTOR, p-p70S6K expression levels were significantly up-regulated in the 3 W group compared to the NS group, confirming that the mTOR pathway was significantly activated during peritoneal fibrosis. RAPA significantly inhibited the phosphorylation of mTOR and p70S6K but did not have significant effects on p-Akt upstream of mTOR. BEZ235 had significant inhibitory effects on all signaling molecules (p-Akt, p-mTOR, and p-p70S6K) in the mTOR pathway.

**Conclusion:** RAPA did not up-regulate p-Akt in a negative feedback fashion. Both drugs effectively inhibited peritoneal fibrosis.

## Background

Long-term peritoneal dialysis will cause peritoneal fibrosis and eventually result in ultrafiltration failure, which is an important cause of technical failure and death in peritoneal dialysis patients [1]. To reduce the development of peritoneal fibrosis and prolong the technical survival of peritoneal dialysis patients, relevant clinical and basic studies have been implemented.

Many studies, including our previous study [2–7], have confirmed that the mammalian target of rapamycin complex 1 (mTORC1)-specific blocker rapamycin (RAPA) inhibits the mesothelial-mesenchymal transition and contributes to the prevention and treatment of peritoneal fibrosis. However, the effect of RAPA in anti-tumor treatment is not ideal, so we worried about whether the anti-peritoneal fibrotic effect of RAPA was reduced. Blocking mTORC1 alone may activate phosphatidyl-inositol 3-kinase/protein kinase B (PI3K/Akt) through the insulin receptor substrate-1 (IRS1) negative feedback loop to attenuate the inhibitory effect on mTORC1 [8]. In addition, specific mTORC1 blockers are not sensitive to mammalian target of rapamycin complex 2 (mTORC2), but mTORC2 can phosphorylate the serine of Akt to continuously activate the PI3K/Akt/mTOR signaling pathway in a feedback fashion [9] and reduce the drug efficacy. Therefore, we sought to find a drug that could more completely block the mTOR pathway and induce more pronounced anti-peritoneal fibrotic effects.

The novel PI3K/mTOR dual-blocker BEZ235 is an anti-tumor drug that has been applied in phase II clinical trials for advanced solid tumors, including breast cancer, prostate cancer, and kidney cancer, as well as leukemia [10]. In this study, we observed the regulation of the mTOR pathway in rats with peritoneal fibrosis by RAPA and BEZ235 to compare the anti-peritoneal fibrotic functions of these 2 different types of mTOR pathway blockers.

## Material and methods

### Major reagents

Chlorhexidine gluconate (CG) was purchased from Sigma-Aldrich (St. Louis, MO, USA). RAPA was purchased from Solarbio (Beijing, China). BEZ235 was obtained from Selleck (Houston, TX, USA). The mouse anti-fibronectin (FN), mouse anti-α-smooth muscle antigen (α-SMA), and mouse anti-collagen 1A1 (Col 1) monoclonal antibodies were purchased from Santa Cruz (Santa Cruz, CA, USA). The rabbit anti-mTOR, rabbit anti-phospho-mTORSer2448, mouse anti-phospho-AktSer437, mouse anti-Akt, mouse anti-phospho-p70S6KThr389, and rabbit anti-p70S6K monoclonal antibodies were purchased from Cell Signaling Technology (Danvers, MA, USA). The immunohistochemistry ultra-sensitive SP reagent kit was obtained from BioTNT (Shanghai, China).

### Experimental animals and grouping

The laboratory animals were purchased from Sippr-BK laboratory animal Co. Ltd (Shanghai, China). A total of 40 clean-grade, male Sprague-Dawley (SD) rats with body weights of ⁓200 g/animal were adaptively housed for 7 d and then randomly divided into five groups with eight animals per group. The normal group (N group) did not receive any intervention for 3 weeks. The normal saline group (NS group) received an intraperitoneal injection of normal saline at 1 mL/100 g daily for 3 weeks. The model group (3 W group) received an intraperitoneal injection of 0.1% CG at 1 mL/100 g daily for 3 weeks. The RAPA group received an intraperitoneal injection of 0.1% CG at 1 mL/100 g + a RAPA gavage at 0.5 mg/d daily for 3 weeks. The BEZ235 group received an intraperitoneal injection of 0.1% CG at 1 mL/100 g + a BEZ235 gavage at 2.5 mg/d daily for 3 weeks.

### Specimen collection

The rats in each group were sacrificed after 3 weeks. An approximately 1 cm^2^ abdominal wall tissue sample was resected by avoiding the injection location. The tissues were fixed in 4% neutral formalin for 24 h and then embedded in paraffin for pathological and immunohistochemistry analyses. The mesenteries were excised, immediately placed in liquid nitrogen, and stored in a −80 °C freezer for protein and RNA extraction.

### Pathological evaluation of the parietal peritoneum

Parietal peritoneum tissues were embedded in paraffin and prepared into sections with a 2 µm thickness. Pathological changes in the peritoneum were observed using Masson’s staining. The peritoneal thickness was observed under an optic microscope (200×). Five fields per specimen were randomly selected, and the thickness of the peritoneal tissues at different locations was measured using the Image-Pro Plus software 6.0 to obtain the mean value.

### Immunohistochemistry analysis of the parietal peritoneum (FN, col 1, and α-SMA)

Paraffin sections were conventionally dehydrated, deparaffinized, and heated for antigen retrieval. The tissues were immersed in solution A of the SP reagent kit for 30 min and then washed with a phosphate-buffered saline (PBS) solution several times. The sections were blocked in solution B for 10 min and then incubated with the primary antibodies (FN 1:400; α-SMA 1:100, and Col 1 1:400) at 4 °C overnight. On the second day, the tissues were immersed in solution C for 30 min and then solution D for 10 min. Immunostaining was performed using the 3,3'-diaminobenzidine tetrahydrochloride (DAB) reagent kit. The cell nuclei were stained with hematoxylin. The sections were washed, dehydrated, and mounted. Five fields per specimen were also randomly selected, and the brown staining area’s cumulative optical density was measured using the Image-Pro Plus software 6.0 to obtain the mean value, as well.

### RT-PCR and real-time PCR

The FN, Col 1, and β-actin mRNA expression levels were measured using quantitative real-time RT-PCR. Total RNA was extracted from the mesentery using the TRIzol reagent (Invitrogen). Reverse transcription was performed using the standard reagent (Promega). Real-time PCR amplification was performed using the SYBR Green master mix (Toyobo) in the Opticon 3 real-time PCR detection system (Bio-Rad). The primers used for FN, Col 1, and β-actin amplification are shown below. The FN upstream primer was 5' AAG GCA ATG GGC GTA TCA C 3', and the downstream primer was 5' TGG GTC TGG GGT TGG TAA AT 3'. The Col 1 upstream primer was 5' CCC AGA CAG AAG TCA TAG CC 3', and the downstream primer was 5' TCA GTT AGC CTT GCC TTT G 3'. The β-actin upstream primer was 5' CCT CTA TGC CAA CAC AGT 3', and the downstream primer was 5' AGC CAC CAA TCC ACA CAG 3'. The real-time RT-PCR conditions were as follows: one cycle of denaturation at 95 °C for 30 s, 40 cycles of denaturation at 95 °C for 5 s and annealing and extension at 60 °C for 30 s, and a final extension at 72 °C for 10 min. β-actin was used as internal control.

### Western blotting analysis

Approximately 100 mg of rat mesentery was added to lysis buffer and homogenized into a homogenate for total protein extraction. The total protein concentration was measured using the bicinchoninic acid (BCA) method. The protein extracts were separated using SDS-PAGE and transferred onto a nitrocellulose membrane. The membrane was blocked in Tris-buffered saline with 0.05% Tween 20 (TBST) containing 5% nonfat milk at room temperature for 1 h and then incubated with primary antibodies at 4 °C overnight (the primary antibody concentrations were as follows: anti-FN 1:500, anti-α-SMA 1:500, anti-mTOR 1:1000, anti-phospho-mTOR^Ser2448^ 1:1000, anti-phospho-AktSer^437^ 1:1000, anti-Akt 1:1000, anti-phospho-p70S6K^Thr389^ 1:1000, anti-p70S6K 1:1000, and anti-β-actin 1:10,000). After washing three times, the membrane was incubated with the secondary antibody at room temperature for 1 h. The membrane was reacted with the electrochemiluminescence (ECL) reagent, exposed to an X-ray film, developed, and fixed. The results were detected using a gel image analysis system on a computer.

### Statistical analysis

The statistical analysis was performed using the SPSS 16.0 statistical software. The data were expressed as the mean ± standard deviation (x ± s). *p* < .05 indicated that the difference was significant. Comparisons between groups were performed using one-way analysis of variance (ANOVA). Pairwise comparisons were performed using the least significant difference (LSD) method.

## Results

### Gross presentations of the mesentery

**Figure 1.**
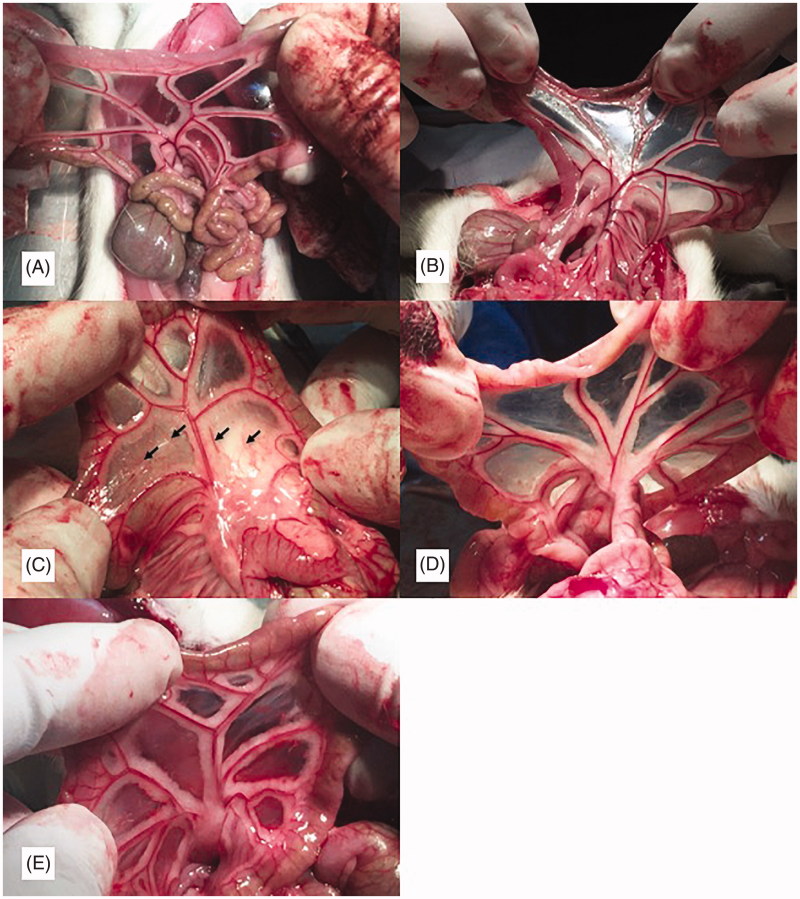
Gross presentations of the rat mesentery. A: N group; B: NS group; C: 3W group; D: RAPA group; E: BEZ235 group : Angiogenesis.

In the 3 W group, the mesentery was significantly thickened, the transparency was decreased, and angiogenesis was observed. The RAPA and BEZ235 groups had thinner mesenteries, higher transparency, and reduced angiogenesis than the 3 W group (Figure 1).

### Masson’s staining of the parietal peritoneum

**Figure 2.**
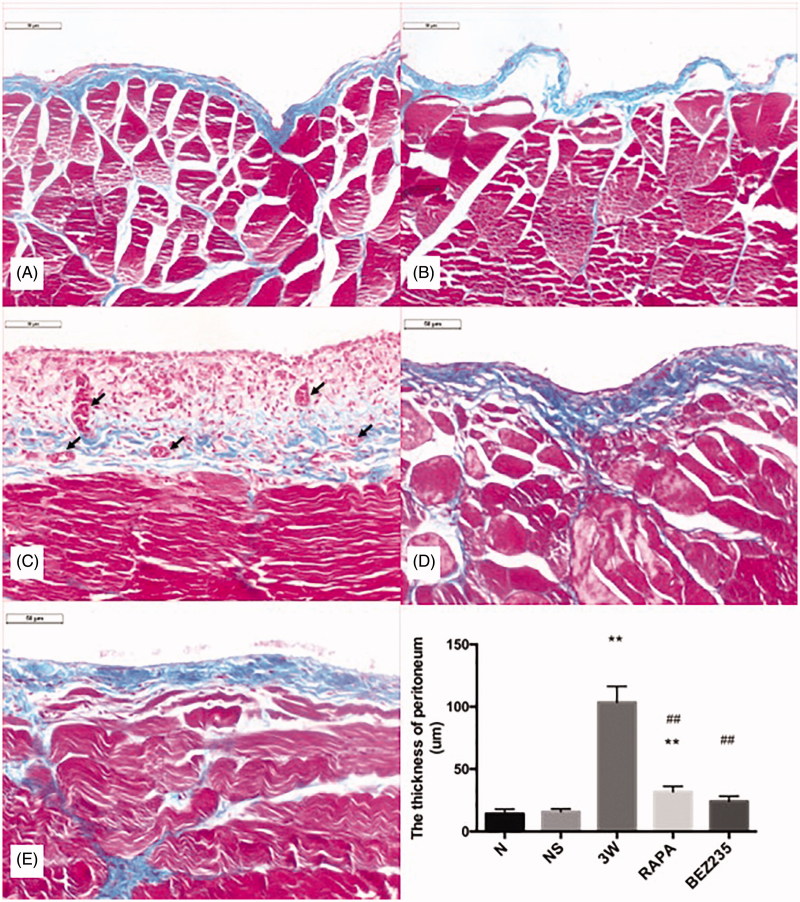
Masson’s staining to evaluate the rat parietal peritoneum (200X). A: N group; B: NS group; C: 3W group; D: RAPA group; E: BEZ235 group. **: vs NS group, *p*<.01; ##: vs 3W group, *p*<.01. : Angiogenesis.

The dense layer of collagen in the parietal peritoneum was thin in the rats in the N and NS groups, and inflammatory cells and blood vessels were rarely observed. In the 3 W group, the dense layer was significantly thickened, the peritoneal thickness was significantly increased compared to the NS group (103.60 ± 12.77 µm vs 15.78 ± 2.28 µm, *p* < .01), and the infiltration of various inflammatory cells and obvious angiogenesis were observed. After intervention with RAPA and BEZ235, the peritoneal thickness significantly thinned, and the inflammatory cell infiltration and angiogenesis significantly decreased (Figure 2).

### FN expression in the parietal peritoneum

**Figure 3.**
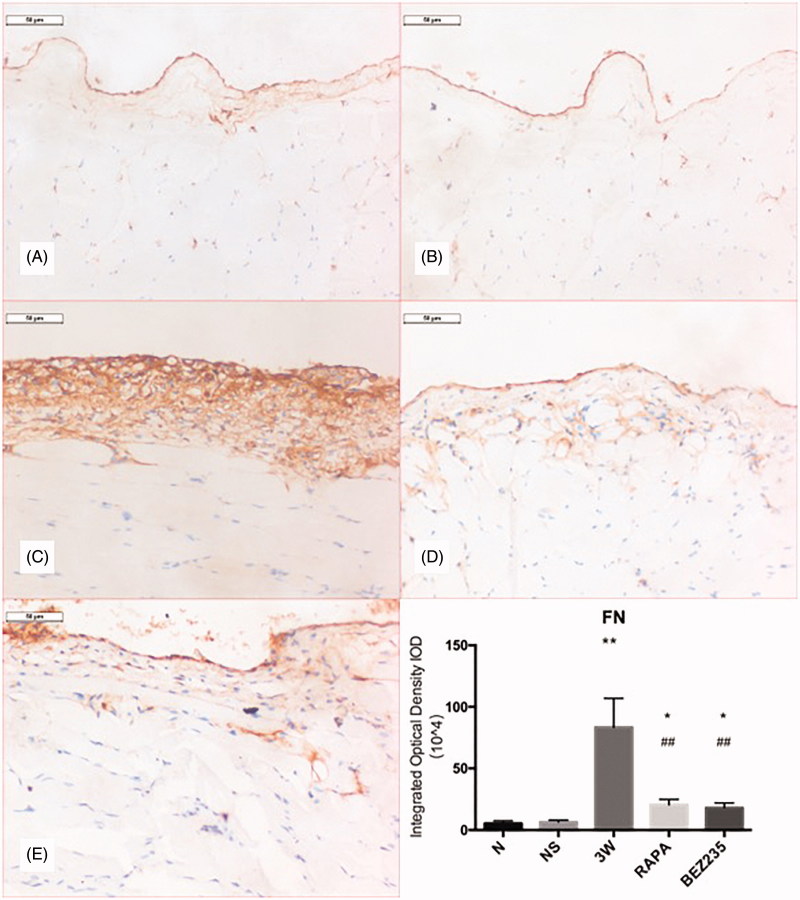
FN expression in the rat parietal peritoneum (200X). A: N group; B: NS group; C: 3W group; D: RAPA group; E: BEZ235 group. *: vs NS group, *p*<.05; **: vs NS group, *p*<.01; ##: vs 3W group, *p*<.01.

The surface of the peritoneum in the N and NS groups exhibited basal linear FN expression levels. In the 3 W group, extracellular matrix deposition was significantly increased, and FN showed a thick zonal distribution along the dense layer. The RAPA and BEZ235 groups had sparse FN expression in the dense layer, and the expression was significantly decreased compared to the expression in the 3 W group (20.41 ± 4.56 × 10^4^/high-power field vs 83.94 ± 23.44 × 10^4^/high-power field, *p* < .01; 18.09 ± 3.87 × 10^4^/high-power field vs 83.94 ± 23.44 × 10^4^/high-power field, *p* < .01) (Figure 3).

### Col 1 expression in the parietal peritoneum

**Figure 4.**
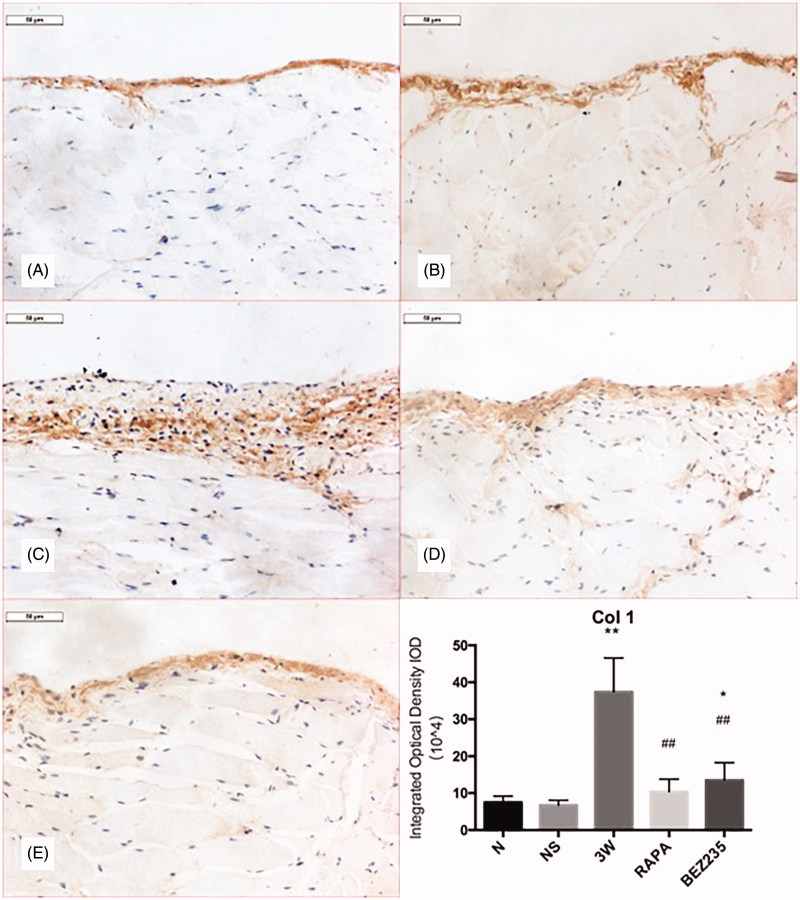
Col 1 expression in the rat parietal peritoneum (200X). A: N group; B: NS group; C: 3W group; D: RAPA group; E: BEZ235 group. *: vs NS group, *p*<.05; **: vs NS group, *p*<.01; ##: vs 3W group, *p*<.01.

Col 1 expression in the N and NS groups showed a thin-stripped pattern. The positive expression area for Col 1 in the 3 W group was significantly widened. Compared with 3 W group, Col 1 expression in the RAPA and BEZ235 groups were significantly decreased (10.41 ± 3.36 × 10^4^/high-power field vs 37.4 ± 9.19 × 10^4^/high-power field, *p* < .01; 13.48 ± 4.78 × 10^4^/high-power field vs 37.4 ± 9.19 × 10^4^/high-power field, *p* < .01) (Figure 4).

### α-SMA expression in the parietal peritoneum

**Figure 5.**
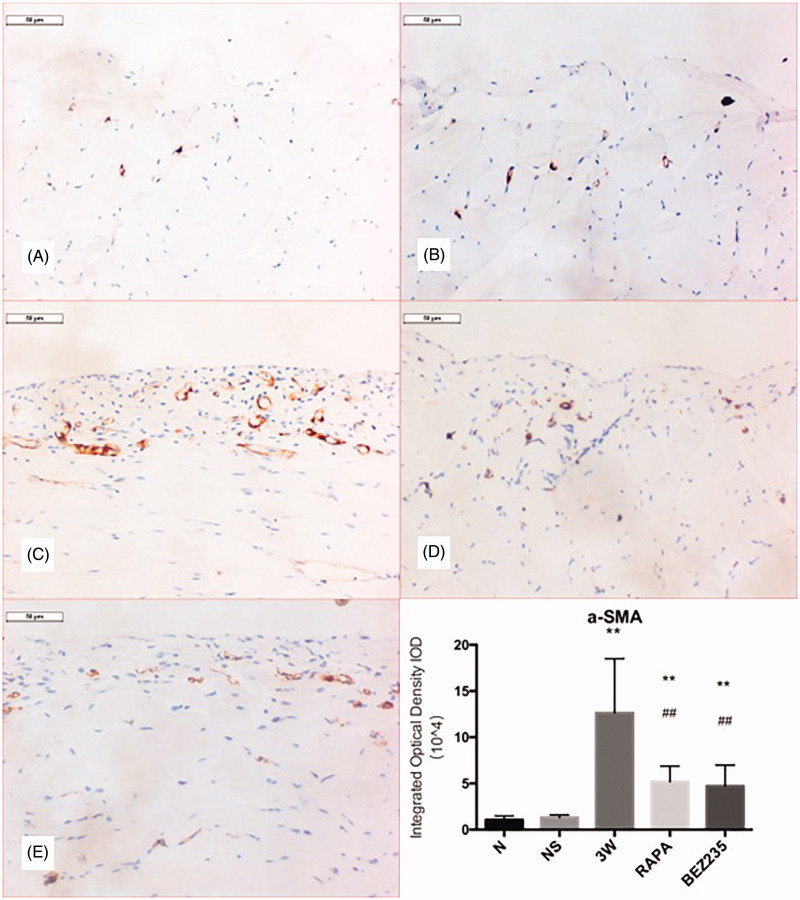
α-SMA expression in the rat parietal perineum (200X). A: N group; B: NS group; C: 3W group; D: RAPA group; E: BEZ235 group. **: vs NS group, *p*<.01; ##: vs 3W group, *p*<.01.

α-SMA-positive cells were occasionally observed in the N and NS groups but were significantly increased in number in the 3 W group. Angiogenesis and inflammatory cell hyperplasia were inhibited in the RAPA and BEZ235 groups, and the numbers of α-SMA-positive cells were significantly decreased compared to the numbers in the 3 W group (5.20 ± 1.67 × 10^4^/high-power field vs 12.66 ± 5.85 × 10^4^/high-power field, *p* < .01; 4.73 ± 2.26 × 10^4^/high-power field vs 12.66 ± 5.85 × 10^4^/high-power field, *p* < .01) (Figure 5).

#### FN and col 1 mRNA expressions in the mesentery

**Figure 6.**
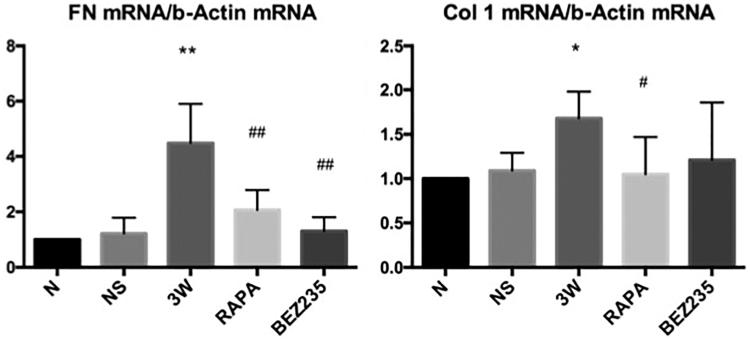
FN and Col 1 mRNA expressions in the rat mesentery. *: vs NS group, *p*<.05; #: vs 3W group, *p*<.05; **: vs NS group, *p*<.01; ##: vs 3W group *p*<.01.

The FN and Col 1 mRNA expression levels in the mesentery of the rats were increased by 4.48-fold and 1.68-fold in the 3 W group compared to the N group, respectively. The FN and Col 1 mRNA expression levels in the 2 drug intervention groups had different degrees of reduction compared to expression in the 3 W group (Figure 6).

#### FN and α-SMA expressions in the mesentery

**Figure 7.**
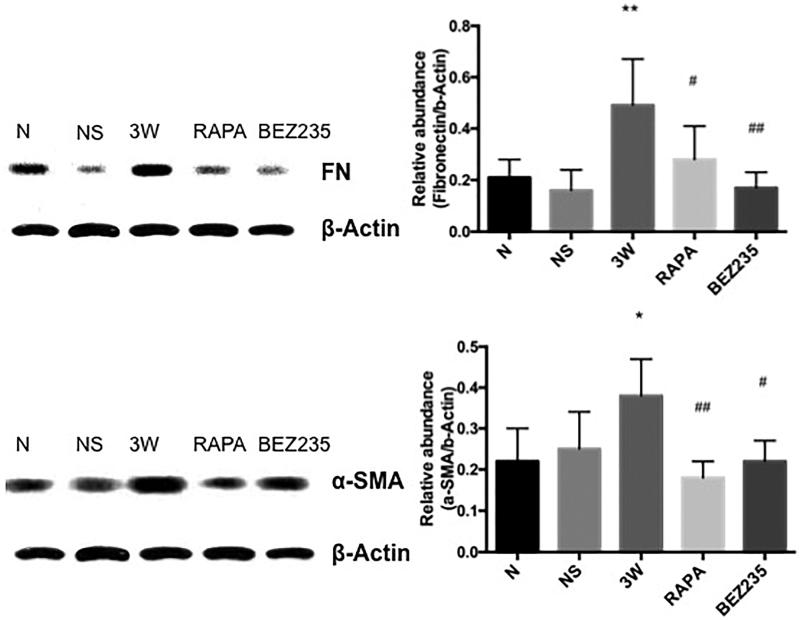
FN and α-SMA expressions in the rat mesentery. *: vs NS group, *p*<.05; #: vs 3W group, *p*<.05; **: vs NS group, *p*<.01; ##: vs 3W group, *p*<.01.

FN and α-SMA expressions in the mesentery were significantly increased in the 3 W group compared to the NS group. After intervention with RAPA and BEZ235, FN (0.28 ± 0.13 vs 0.49 ± 0.18, *p* < .05; 0.17 ± 0.06 vs 0.49 ± 0.18, *p* < .01) and α-SMA (0.18 ± 0.04 vs 0.38 ± 0.09, *p* < .01; 0.22 ± 0.05 vs 0.38 ± 0.09, *p* < .05) expressions were significantly decreased compared to the expression levels in the 3 W group, whereas no significant difference was found between these 2 treatment groups (Figure 7).

#### Expressions of the mTOR pathway components (p-Akt, p-mTOR, and p-p70S6K) in the mesentery

**Figure 8.**
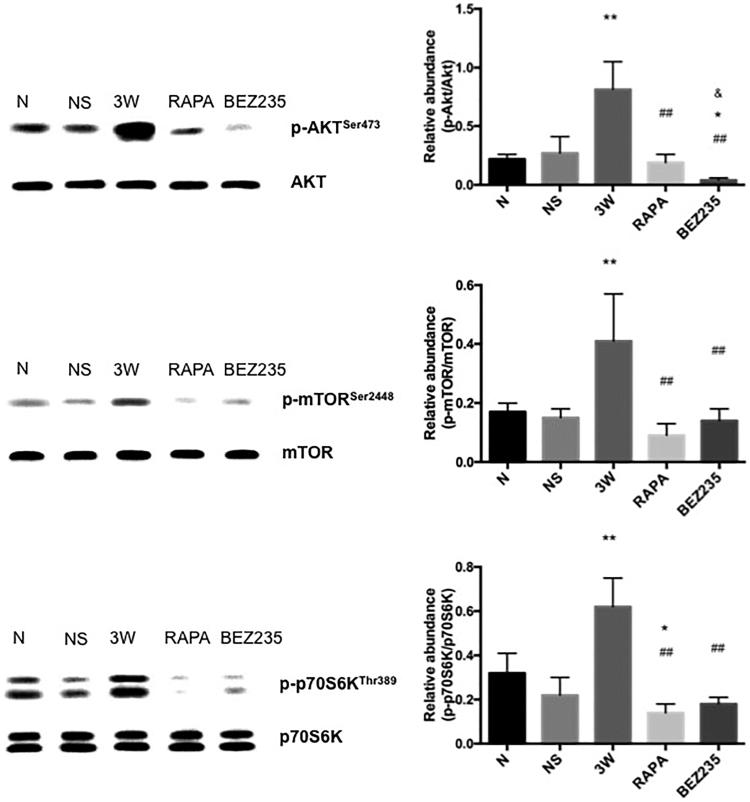
Expressions of the mTOR pathway components in the rat mesentery. *: vs NS group, *p*<.05; **: vs NS group, *p*<.01; ##: vs 3W group, *p*<.01; &: vs RAPA group, *p*<.05.

The p-Akt, p-mTOR, and p-p70S6K expression levels were significantly increased in the 3 W group compared to the expression levels in the NS group. The p-Akt, p-mTOR, and p-p70S6K expression levels were significantly decreased in the RAPA group compared to the expression levels in the 3 W group; however, p-Akt expression did not significantly differ between the RAPA and NS groups. Additionally, the mTOR pathway was significantly inhibited in the BEZ235 group, and p-Akt down-regulation was more obvious in this group than in the RAPA group (0.04 ± 0.02 vs 0.19 ± 0.07, *p* < .05) (Figure 8).

## Discussion

TGF-β1 is the most important initiation factor of peritoneal fibrosis [11]. This factor can activate both the Smad and the non-Smad signaling pathways simultaneously. The pro-fibrosis function of the TGF-β/Smad signaling pathway has been recognized, and this pathway extensively participates in the fibrotic process in the kidney, heart, liver, and peritoneum [12,13]. However, the TGF-β/Smad pathway is not sufficient to explain the whole peritoneal fibrotic process. Through ‘crosstalk effects’ with other signaling pathways, TGF-β can activate signaling pathways other than Smad, including the protein kinase C (PKC) pathway [14], the extracellular signal-regulated kinase (ERK) pathway [15], and the phosphatidyl-inositol-3-kinase/serine-threonine-specific protein kinase (PI3K/Akt) pathway [16]. Patel et al. [16] showed that whether Smad knockout or not, the PI3K/Akt expression in mice peritoneum would be up-regulated after the transfection with an adenovirus expressing TGF-β1mTOR is located downstream of PI3K/Akt. This enzyme has serine/threonine (Ser/Thr) protein kinase activity and is a central regulatory factor in cell growth and metabolism [17]. mTOR has 2 forms: mTORC1 and mTORC2 [18]. The interaction between mTORC1 and the FK506-binding protein 12-rapamycin (FKBP12-RAPA) inhibits mTOR kinase activity [19]. The most important downstream target protein of mTORC1 is the 4E-binding protein 1 (4E-BP1) and the 70-kDa ribosomal protein S6 kinase (p70S6K) [20,21]. Non-phosphorylated 4E-BBP1 binds to the eukaryotic translation initiation factor 4E (eIF4E) and inhibits its activity, whereas 4E-BP1 phosphorylated by mTOR dissociates from eIF4E and loses its inhibitory function on eIF4E, thereby initiating mRNA translation [21]. mTORC1 can also phosphorylate p70S6K to initiate ribosomal protein translation.

RAPA interacts with the FK506-binding protein to form a complex that binds to mTORC1 to reduce the synthesis of cell cycle-associated proteins so as to arrest the cells at the G1 phase [22]. This drug is mainly applied for anti-rejection in kidney transplantation, drug-eluting stent, and anti-tumor treatment patients in the clinic. Due to its excellent functions, including anti-cell proliferation, reduction of extracellular matrix (ECM) accumulation, and inhibition of angiogenesis, RAPA has been gradually applied in the anti-fibrosis field. Haller et al. [23] confirmed that RAPA significantly attenuated cardiac fibrosis in rats with uremic cardiomyopathy. Yin et al. [24] showed that treatment of transgenic mice over-expressing kidney injury molecule 1 with RAPA significantly reduced serum creatinine and inhibited renal fibrosis. Our group previously applied RAPA intervention in rats with peritoneal fibrosis [2]. The results confirmed that RAPA significantly inhibited peritoneal fibrosis. Subsequently, Sağıroğlu et al. [6] used RAPA to treat rats with peritoneal fibrosis and showed that only 75% of the rats in the RAPA group had mild peritoneal fibrosis, whereas 100% of the rats in the control group had different degrees of peritoneal fibrosis. *In vivo* and *in vitro* studies by González et al. [4] showed that RAPA reduced the endothelial-to-mesenchymal transition and inhibition of the formation of capillaries or lymphatic vessels to prevent the development of type 1 ultrafiltration failure.

Although RAPA has excellent anti-fibrosis functions, very few clinical trials have obtained successful anti-tumor treatment results. The major reason is that blocking mTORC1 alone will activate PI3K/Akt upstream of mTORC1 through the IRS-1 negative feedback loop to attenuate the inhibitory effect on mTORC1 [8]. Furthermore, RAPA is not sensitive to mTORC2, and mTORC2 can phosphorylate the serine of Akt to activate the PI3K/Akt/mTOR pathway through a feedback loop [9]. To address the above issue, novel mTOR blockers capable of blocking many sites have been developed. As a PI3K/mTOR dual-blocker, BEZ235 can simultaneously function on the active sites of these 2 kinases to avoid p-Akt up-regulation through the mTORC1-S6K-IRS1 negative feedback loop. Phase I clinical trials on solid tumors and blood system tumors have confirmed that BEZ235 has excellent anti-cell proliferation functions [25,26]. BEZ235 can also reduce VEGF-induced angiogenesis [27]. Currently, one study has applied BEZ235 in the anti-fibrosis field [28]. In this study, we aimed to determine the anti-peritoneal fibrotic function of BEZ235.

According to our previous experience [2], the method of IP peritoneal dialysis fluid to establish peritoneal fibrosis model has a lot of defects, such as: poor tolerance to the rats, difficult to operate and a few deaths. In contrast, the method of IP CG is easier to operate and has a higher success rate. Based on these reasons, we abandoned the previous method.

Observation of the parietal peritoneum using Masson’s staining showed that the pathological presentations of fibrosis, including collagen deposition under mesothelial cells, inflammatory cell infiltration, and capillary angiogenesis, were significantly attenuated in the two drug intervention groups compared to the model group. The RT-PCR, immunohistochemistry and western blotting results showed that the FN, Col 1 mRNA, and FN, Col 1, α-SMA expressions were significantly decreased in both the RAPA and BEZ235 groups to the levels in the model group; however, no significant difference was found between these two groups. The above experiments confirmed that RAPA and BEZ235 both effectively inhibited peritoneal fibrosis and that the anti-fibrotic effects between these two drugs did not significantly differ.

During anti-tumor treatment, blocking mTORC1 with RAPA will up-regulate PI3K/Akt activity upstream of mTORC1 through the IRS-1 negative feedback loop [8]. Therefore, we performed western blotting to observe the regulatory function of these two drugs on the mTOR pathway. As the functional sites and downstream signaling molecules of these two drugs, p-mTOR and p-p70S6K both had significantly reduced expression levels. BEZ235 belongs to the PI3K/mTOR dual-blockers and can block PI3K upstream of Akt and mTOR downstream of Akt simultaneously. RAPA belongs to the mTORC1-specific blockers and can only function on mTORC1 downstream of Akt. Therefore, p-Akt expression was significantly decreased in the BEZ235 group. In contrast, the p-Akt expression level did not significantly differ between the RAPA and NS groups, and RAPA did not induce p-Akt up-regulation through a negative feedback loop. Novalic et al. [29] applied RAPA to treat a polycystic kidney disease mouse model with loss of Pkd1. The results showed that neither the treatment time nor the RAPA dose affected p-Akt expression, whereas p-S6 expression was negatively correlated with the RAPA dose. Finally, the development of renal cysts was only associated with p-S6 expression. We did not observe p-Akt up-regulation, suggesting that the negative feedback mechanism present in tumor cells was very mild in non-tumor cells. Although RAPA and BEZ235 regulated p-Akt differently, the p-p70S6K down-regulation and anti-peritoneal fibrotic functions were basically similar between these two groups. According to the experimental results in this study and the work by Novalic et al. [29], we considered that the final pathological changes were only associated with p-70S6K expression and were not significantly associated with p-Akt expression.

The study results showed that the anti-peritoneal fibrotic function of RAPA was not worse than that of BEZ235. In addition to direct inhibition of cell proliferation, RAPA might have other anti-fibrotic mechanisms. (1) Gui et al. [30] applied RAPA to treat mice with bleomycin-induced pulmonary fibrosis and found that RAPA attenuated pulmonary fibrosis through the promotion of autophagy. (2) Sekiguchi et al. [31] reported that RAPA inhibited peritoneal fibrosis in mice induced by an adenovirus expressing TGF-β through reduction of hypoxia-inducible factor-1α (HIF-1α) expression. (3) Xiang et al. [3] proposed that RAPA suppressed the mesothelial cell transition and peritoneal fibrosis through blocking the Rho GTPase. In future works, we will keep exploring the related mechanisms of mTOR blockers in treating peritoneal fibrosis.

We have to point out that there are still a few limitations in this study. Because the results of α-SMA mRNA and Col 1 didn’t perform optimally, they were excluded from Figure 6 and 7. However, we don’t think this would invalidate our findings. In future research, we will complete our work more perfectly.

In summary, the mTORC1-specific blocker did not induce p-Akt up-regulation through negative feedback, and RAPA and BEZ235 both significantly down-regulated the mTOR pathway to inhibit the development and progression of peritoneal fibrosis. Both drugs have very broad application prospects in the anti-peritoneal fibrosis field; however, elucidating the specific mechanisms requires further study and investigation.
